# Brazil has a problem: where is its research on eating disorders?

**DOI:** 10.47626/1516-4446-2024-3831

**Published:** 2024-11-25

**Authors:** Jônatas de Oliveira, Thais Santos, João Ferro, Isis Stelmo

**Affiliations:** 1 Faculdade de Medicina, Universidade de São Paulo, São Paulo, SP, Brazil; 2 Instituto de Pesquisa em Comportamento e Comida, São Paulo, SP, Brazil; 3 Universidade Federal de São Paulo, São Paulo, SP, Brazil

It is with a mixture of surprise and concern that we recognize the scarcity of research on eating disorders (EDs) in Brazil. An analysis of the state of affairs about publications on the topic is required to facilitate further comprehension of research pathways, factors that influence these trajectories, and funding sources. Care processes and individual experiences in the public healthcare system are also a cause for concern, due to the lack of materials, training programs, and public education initiatives on EDs that could enable early identification and subsequent care.

When Kolar et al.^1^ reported ED prevalence rates for Latin America, including Brazil, they did not consider several studies published in our country. Their systematic review and meta-analysis involved sample sizes of around 200 individuals and indicated a mean point-prevalence rate of 0.1% for anorexia nervosa, 1.16% for bulimia nervosa, and 3.53% for binge-eating disorder in Brazil. In a follow-up study, Kolar and Mebarak^2^ reported prevalence rates 0.04% to 0.09% for anorexia nervosa and 0.13% to 0.27% for bulimia nervosa. Additionally, three recent Brazilian studies identified a general point-prevalence of 0.40% for EDs in children aged 6-14 years, and a point-prevalence of 0.7% for bulimia nervosa, 1.4% for binge-eating disorder, and 6.2% for recurrent binge eating.^2^

In 2020 Moser et al.^3^ published a Brazilian version of the Eating Disorder Examination Questionnaire, the most widely used instrument to assess ED symptoms. First published in 1994, it has good psychometric and diagnostic properties in other languages and populations. While abbreviated versions and other semi-structured clinical interviews based on it have been developed abroad, despite being cited 17 times since its publication, the Brazilian Eating Disorder Examination Questionnaire has yet to be validated in national samples of people with ED. It is widely-known that a substantial component of the validation process for these questionnaires is the availability of a significant group of formally diagnosed patients, which is essential for evaluating a questionnaire’s psychometric properties and is necessary for determining whether a scale developed abroad is suitable for local sociocultural nuances.

While international research offers valuable insights, it is imperative to consider local sociocultural diversity, which can influence ED presentation and treatment. Besides the need for additional epidemiological national studies, such as that of Kolar et al.,^1^ Brazilian research is frequently grounded in etiological models and understanding of eating behavior proposed in the global north. Thus, a more specific and sensitive approach is needed for our distinctive cultural characteristics, regional differences, and ethnic and social diversity.

Moreover, Wade et al. published a research program highlighting the necessity of integrated interventions when co-occurring mental health conditions hinder effective ED treatment,^4^ which included a comprehensive four-step protocol encompassing three broad intervention approaches: alternate, modular, and transdiagnostic. What would be the estimated time frame for conducting similar research in Brazil in comparable terms, given our country's characteristics and size?

Why have other regions of the world made more progress in the study of EDs? While a significant body of Brazilian research exists on other mental disorders, including advanced diagnostic technology and randomized controlled trials, training on ED diagnosis and treatment is very limited in both public and private settings. As far as public universities are concerned, on the one hand other psychiatric disorders have received more attention, resulting in a multitude of studies and dedicated personnel for academic training, teaching, and research, which has consequently improved the quality of medical education. On the other hand, not all psychiatry residency programs have the necessary resources to teach students about EDs. Thus, knowing that that access to complex data on mental health phenomena depends exclusively on scientific advancements a country has made in that specific field, why is there such a scarcity of publications on EDs in Brazil?

To illustrate the paucity of national research on EDs, we searched the *Brazilian Journal of Psychiatry* and the *International Journal of Eating Disorders,* both international journals, to determine whether Brazilian researchers have successfully contributed to the national data on EDs, but we found few results. Since various methods can be employed to examine this phenomenon, we are making efforts on multiple fronts to find different outcomes. We have also been considering an investigation into the personal journeys of national public health system patients to highlight the population’s reception in the health system and their initial ED screening experiences.

In recent volumes of the *Brazilian Journal of Psychiatry*, Brazilian research has encompassed a wide range of mental health conditions, including depression, bipolar disorder, obsessive-compulsive disorder, and substance use disorders. However, the most recent publication on EDs with a Brazilian author was in volume 43 (2021), in which a Brazilian researcher co-authored a single editorial on ED risk factors, discussing the latest research and its implications.^5^ Upon reviewing earlier issues, it became evident that we are still in the early stages of cross-cultural adaptation, particularly concerning validation studies in clinical samples. For instance, the Brazilian version of the SCOFF questionnaire was validated in a university population in 2021.^6^ Furthermore, we encountered a single review, in which only one study had been conducted by a Brazilian research group: an assessment of the risk of comorbid EDs in individuals with attention-deficit/hyperactivity disorder.^7^

Nevertheless, recent prevalence data for bulimia nervosa, binge eating disorder, and recurrent binge eating were published from a robust two-phase in-person household survey conducted in Rio de Janeiro between 2019 and early 2020 (prior to the pandemic), which initially screened 2,297 individuals, followed by structured clinical interviews with 305 participants.^8^ The majority of adolescents with EDs are high-income White girls, but the prevalence is increasing among boys, and the average age of patients is reducing.^9^ In countries with more developed research models, more relevant aspects are considered, resulting in a deeper understanding of EDs, although in Brazil we are still characterizing clinical samples with fewer population studies.^1^,^8^

Our research group has been working on a national systematic review, whose initial analysis resulted in five themes: demographic and socioeconomic profiles, clinical and dental impacts, psychological and behavioral aspects, family and social relationships, and pharmacological treatments and assessment, which indicates that there has been insufficient investment, technology, and action in this area. Future analyses will uncover the trajectory of research and the contributions of individuals and institutions. We have made significant progress toward understanding ED symptoms, thanks to an elegant investigation.^10^ However, as the authors pointed out, “further research should investigate whether positive screenings translate to actual ED diagnoses.”

Given this scenario, it is imperative to encourage ED research among Brazilian researchers, study centers, outpatient clinics, and health care facilities in the national health system. Future challenges include updated tools and methods and the validation of scales necessary for screening and diagnosing on a national level. Such efforts will require partnerships, resources, and collaboration with the Brazilian public health system. Significant effort and energy are required to provide quality data on the characteristics of Brazilians with EDs. Bibliometric analyses can be useful for tracking and identifying which graduate programs and researchers have contributed the most, and how they have contributed to the improvement of diagnostic technologies, team training, participant recruitment, and knowledge production.

Effort should be concentrated on upgrading public health services and instructing health professionals, given that a large part of the population cannot afford private treatment. Therefore, we recognize the importance and usefulness of prevalence and characterization studies based on a more comprehensive and nuanced approach than that employed in previous studies based exclusively on international models. However, there are more pressing matters, such as developing national etiological models and conducting studies that account for the diversity of the country’s population and its cultural needs, as well as the implications of these differences regarding the onset and presentation of EDs. Addressing this gap is essential for developing effective public health policies and culturally appropriate intervention strategies that can improve the health and well-being of the people affected by these challenging disorders.

## Disclosure

The authors report no conflicts of interest.
